# MOTIVATION AS A MECHANISM: THE LONGITUDINAL RELATIONSHIP BETWEEN PERSONAL RESOURCES AND ACTIVITY ENGAGEMENT

**DOI:** 10.1093/geroni/igz038.2994

**Published:** 2019-11-08

**Authors:** Allura F Lothary, Tara L Queen, Thomas M Hess

**Affiliations:** 1 North Carolina State University, Raleigh, North Carolina, United States; 2 University of North Carolina at Chapel Hill, Carrboro, North Carolina, United States

## Abstract

Past research has demonstrated an association between health and cognitive resources, intrinsic motivation, and activity participation in older adulthood, both cross-sectionally and from a daily perspective (e.g., Queen & Hess, 2018; Hess et al., 2018). This highlights the potential importance of motivation as a mediator of the impact of changing personal resources on engagement in cognitively beneficial activities. This study expanded on prior research by examining these relationships longitudinally in a large representative sample of adults over 50. Specifically, we used data from the Health and Retirement Survey (N = 5600) to create two 4-year longitudinal assessments, with multi-level structural equation modeling used to test the mediating role of motivation on everyday activity engagement. Consistent with expectations from Selective Engagement Theory (Hess, 2014), motivation served as a partial mediator of the impact of changing resources on engagement, with the effect being selective based on the cognitive demands of the activity.

